# PD-1/PD-L1 Control of Antigen-Specifically Activated CD4 T-Cells of Neonates

**DOI:** 10.3390/ijms24065662

**Published:** 2023-03-16

**Authors:** Christiane Majer, Holger Lingel, Aditya Arra, Hans-Gert Heuft, Dirk Bretschneider, Silke Balk, Katrin Vogel, Monika C. Brunner-Weinzierl

**Affiliations:** 1 Department of Experimental Pediatrics, Medical Faculty, Otto-von-Guericke-University, 39120 Magdeburg, Germany; 2 Institute of Transfusion Medicine and Immunohematology, Medical Faculty, Otto-von-Guericke-University, 39120 Magdeburg, Germany; 3 Maternity Clinic, Hospital Sankt Marienstift, 39110 Magdeburg, Germany

**Keywords:** T-helper cells, CD4 T cell, immune checkpoint molecule, PD-1/PD-L1, *Staphylococcus aureus* (*S. aureus*), bacteria, staphylococcus enterotoxin B (SEB), pediatric immunology, newborn

## Abstract

Newborns are highly susceptible to infections; however, the underlying mechanisms that regulate the anti-microbial T-helper cells shortly after birth remain incompletely understood. To address neonatal antigen-specific human T-cell responses against bacteria, *Staphylococcus aureus* (*S. aureus*) was used as a model pathogen and comparatively analyzed in terms of the polyclonal staphylococcal enterotoxin B (SEB) superantigen responses. Here, we report that neonatal CD4 T-cells perform activation-induced events upon *S. aureus*/APC-encounter including the expression of CD40L and PD-1, as well as the production of Th1 cytokines, concomitant to T-cell proliferation. The application of a multiple regression analysis revealed that the proliferation of neonatal T-helper cells was determined by sex, IL-2 receptor expression and the impact of the PD-1/PD-L1 blockade. Indeed, the treatment of *S. aureus*-activated neonatal T-helper cells with PD-1 and PD-L1 blocking antibodies revealed the specific regulation of the immediate neonatal T-cell responses with respect to the proliferation and frequencies of IFNγ producers, which resembled in part the response of adults’ memory T-cells. Intriguingly, the generation of multifunctional T-helper cells was regulated by the PD-1/PD-L1 axis exclusively in the neonatal CD4 T-cell lineage. Together, albeit missing memory T-cells in neonates, their unexperienced CD4 T-cells are well adapted to mount immediate and strong anti-bacterial responses that are tightly controlled by the PD-1/PD-L1 axis, thereby resembling the regulation of recalled memory T-cells of adults.

## 1. Introduction

Infections are a main cause of neonatal mortality and are primarily of bacterial origin [1]. *Staphylococcus aureus* (*S. aureus*) is a well-known human commensal bacterium carried by 19% of the total population [2]. At the same time, *S. aureus* is also the most common pathogen of skin and soft tissue infections and some invasive infections such as osteomyelitis, sepsis, infectious endocarditis, pneumonia, eye infections and infections of the central nervous system in newborns, infants and children [3]. Hence, *S. aureus* is also one of the most common organisms isolated from newborns and children with health care-associated infections [4]. Every year, these early infections cause more than half a million newborn deaths. Although antibiotics can be used against bacteria, it is becoming increasingly evident that antibiotic-resistant bacteria are rapidly increasing [5]. In addition, antibiotic therapy during the postnatal period is particularly damaging to the development of the immune system when the gut microflora builds up, probably with lifelong consequences [6,7,8,9,10]. In order to support the neonatal immune system to efficiently fight off bacteria by strengthening the body’s defense mechanisms, a better understanding is needed. 

Starting at birth, the immune system already has to defend the organism against previously unknown pathogens and at the same time it has to prevent the body from being harmed by an excessive immune response. The misregulation of either ability inevitably leads to pathogenesis, and often death. Currently, it is well accepted that the immune system of children, especially that of infants and neonates, is different from that of adults, and it is becoming increasingly apparent that these T-cells are well-adapted to what is needed during the period of neonatal life [11]. At birth, neonates hardly have antigen-experienced T-cells and are confronted with environmental antigens in excess. Thus, T-cell differentiation has to be cautiously initiated as it might be at the expense of forming memory. In this respect, fetal and neonatal T-cells are suggested to be derived from different progenitors that have a bias to differentiate into Treg cells [12]. Indeed, even farm animal exposure during pregnancy can induce already regulatory T-cells [13]. The hyporesponsiveness of neonatal naïve CD4 T-cells has been described as being caused by an enhanced Ca^2+^-influx upon TCR triggering, but reduced Erk phosphorylation and a different susceptibility to costimulation [14]. The enhanced TCR sensitivity might be caused by mir181 up-regulation [11,15]. Furthermore, the hyperreactivity of Th2-like-responses has been reported at a molecular level [16]. In addition, a global mitogen-stimulation did not show a Th2 bias, indeed, neonatal T-cells can generate strong Th1 responses—at least in response to mitogens [17]. Signatory cytokines of T-cells have been reported for the neonatal period, such as IL-8, IL-10, IL-17 and IL-22 [18,19,20]. However, only IL-17 has so far been confirmed after antigen-dependent activation, while other studies have been conducted using polyclonal, TCR-unspecific stimulation [21]. Therefore, the full potential of antigen-stimulated neonatal CD4 T-cells is still not known. 

Among the immunoregulatory molecules that modulate T-cell responses, the surface receptor PD-1 and its ligand PD-L1 have emerged as therapeutically targeted immune-checkpoints. PD-1 is inducibly expressed on T-cells providing co-inhibitory signals and thus, pathogens could exploit its pathway to evade the host defense mechanisms [22]. In neonates the PD-1/PD-L1 axis is supposed to be involved in the regulation of inflammation resolution [23]. Further, PD-1 is implicated in the differentiation of exhausted CD4 T-cells in chronic infections. In this setting, the blockade of PD-1/PD-L1 predominantly affects less terminally differentiated T-cell populations [24]. However, the role of the PD-1/PD-L1 axis in regulating neonatal CD4 T-cells still remains incompletely understood. Therefore, examining and exploiting the effects of the PD-1/PD-L1 pathway on antigen-specifically activated neonatal CD4 T-cells could help to boost the immune response against bacteria at a young age in a targeted and developmentally appropriate manner.

Here, a model for human antibacterial T-cell responses was applied [21,25] that allowed us to decipher the characteristics of human neonatal T-cell responses and differentiation in an antigen-specific manner. The antibody mediated blockade of the co-inhibitory receptor PD-1 and its ligand PD-L1 revealed an immediate and tight control of neonatal T-helper cell responses by these immune-checkpoint molecules. Thus, unlocking the control of immune checkpoint molecules showed the full potential of neonatal T-cells, including the co-expression of multiple cytokines.

## 2. Results

### 2.1. Neonatal CD4 T-Cells Show Widespread Activation by S. aureus and SEB 

**Figure 1:**
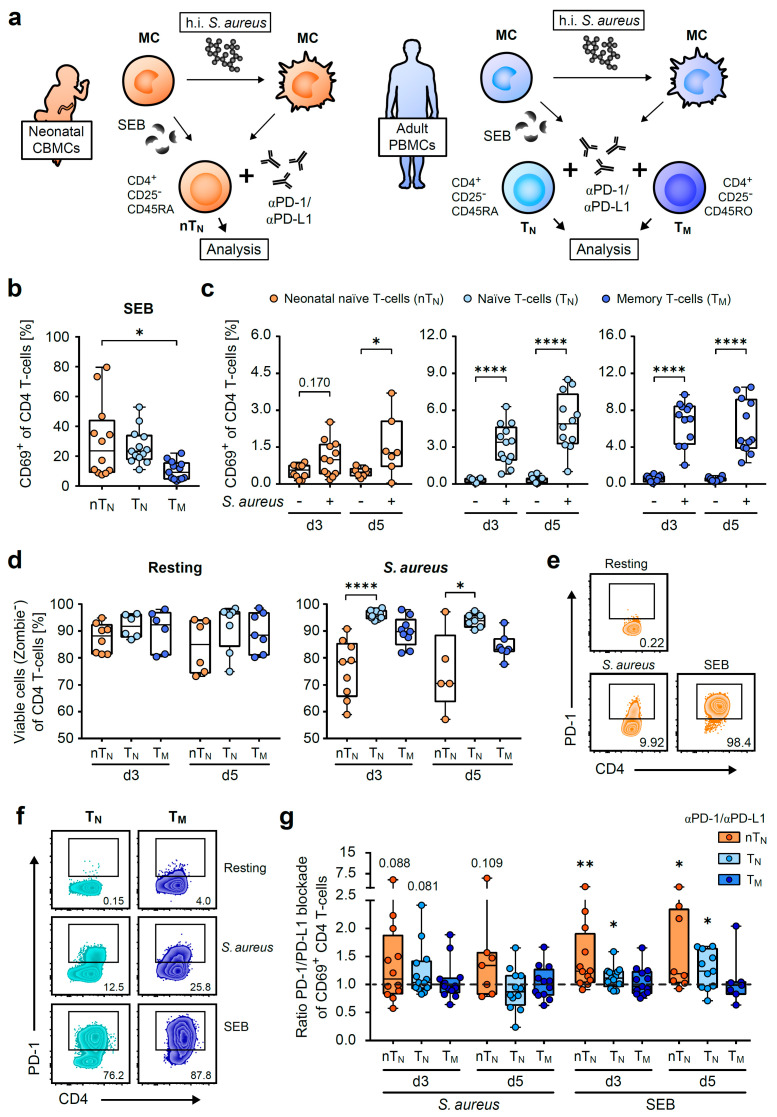
Neonatal CD4 T-cells up-regulate PD-1 and control strong responses against SEB via the PD-1/PD-L1 axis. (**a**) Scheme of activation of neonatal naïve (nT_N_) or adult naïve (T_N_) and memory (T_M_) CD4 T-cells by *S. aureus* or SEB-loaded autologous monocytes (MC). Cells were isolated from neonatal umbilical cord blood mononuclear cells (CBMCs) or adult peripheral blood mononuclear cells (PBMCs), respectively. (**b**,**c**) CD69 expression (day 3) on nT_N_, T_N_ or T_M_ in response to SEB (**b**) or to *S. aureus* vs. resting (**c**). (**d**) Viability of nT_N_, T_N_ or T_M_ under resting conditions (left) or *S. aureus*-induced activation (right). (**e**,**f**) PD-1 expression of nT_N_ (**e**), T_N_ or T_M_ (**f**) on day 5. (**g**) Ratios of CD69 expression of *S. aureus* or SEB-stimulated PD-1/PD-L1-blocked CD4 T-cells to their isotype controls. The significance between blockade and isotype control is depicted above the respective box plot. Data points represent donors in box plots with median, interquartile, and range. Numbers indicate percentages of PD-1-expressing T-cells or *p*-values; * *p* < 0.05, ** *p* < 0.01, **** *p* < 0.0001; *p*-values were calculated using the Kruskal–Wallis test (**b**,**d**), one-way ANOVA with Sidak’s multiple comparison test (**c**), the Wilcoxon test or the paired *t*-test (**g**).

Since neonatal CD4 T-cells react differently from adult T-cells due to age-specific requirements [6,12,14,16,26], we first assessed whether neonatal CD4 T-cells can react antigen-specifically against heat-inactivated (h.i.) *Staphylococcus aureus* (*S. aureus*) bacteria in comparison to the naïve or memory CD4 T-cells of adults. Besides, the common superantigen staphylococcus enterotoxin B (SEB) of *S. aureus* was analyzed separately, and the *S. aureus* SEB^neg^ strain was used to follow the antigen-specificity of T-cell responses [27]. For this purpose, we isolated and activated naïve T-cells from the umbilical cord blood of neonates, as well as T-cells from the peripheral blood of healthy adult donors (Figure 1a). Since a preference for producing regulatory T-cells has been described in the fetus and newborn, CD25^+^ T-cells were routinely depleted by MACS [12]. To specifically compare the properties of antigen-inexperienced CD4 T-cells, the naïve (CD45RA) CD25^neg^ CD4 T-cells of neonates (nT_N_) and adults (T_N_) were enriched in addition to the memory (CD45RO) CD25^neg^ CD4 T-cells (T_M_) using MACS [14]. The enriched CD4 T-cells were treated with PD-1- and PD-L1-blocking antibodies or isotype controls and were either allowed to rest or stimulated with SEB or with *S. aureus*-loaded autologous monocytes and analyzed 3 and 5 days after the onset of stimulation, respectively (Figure 1a) [21,25]. An up-regulation of the TCR-signaling induced surface molecule CD69 was monitored upon the SEB and *S. aureus* encounter but not in the resting conditions of co-cultures of CD4 T-cells with unloaded monocytes (Figure 1b,c). Notably, the frequency of CD69-expressing T_N_ remained higher than that of T_M_ on day 3, when it is likely to already be downregulated (Figure 1b). With a maximum of 80% of CD69-expressing T-helper cells in response to SEB and 3.7% in response to *S. aureus*, the neonatal CD4 T-cells showed a compelling anti-bacterial response that was, in terms of antigen-specific activation, also visible to a greater extent in the adult T-cells (Figure 1b,c). When comparing T-cell viability in resting and activated CD4 T-cells using Zombie staining, similar behavior of the resting T-cells but reduced percentages of viable cells could be detected in the neonatal T-cells only upon activation with *S. aureus*, suggesting activation-induced cell death (AICD) (Figure 1d).

Next, the expression of the surface-molecule PD-1 was determined (Figure 1e,f). Both the SEB and *S. aureus*-induced activation of the neonatal and adult CD4 T-cells led to the strong surface expression of the co-inhibitory receptor. To determine its functionality, we blocked PD-1 and its ligand PD-L1 using specific antibodies or an isotype control and analyzed their impact on activation-induced CD69 up-regulation (Figure 1g). Indeed, the ratio of *S. aureus*-stimulated cells with the isotype or PD-1/PD-L1 blockade revealed that the enhanced frequency of CD69 expression on naïve T-cells was strongly (*p* < 0.05) controlled by the PD-1/PD-L1 axis in response to SEB, and this trend (*p* < 0.09) was similar upon *S. aureus*-mediated stimulation (Figure 1g). This implies that the T-cell activation of naïve T-cells, in particular, remains under PD-1 control. 

### 2.2. PD-1 Controls the Capacity of S. aureus-Specific Proliferative Responses of Neonatal CD4 T-Cells

To investigate the age-related effects of the PD-1/PD-L1 blockade on the proliferative capacity of neonatal and adult CD4 T-cells, naïve and memory CD4 T-cells were CFSE-labelled and incubated with anti-PD-1 and anti-PD-L1 antibodies or an isotype control prior to priming with SEB or h.i. *S. aureus* SEB^neg^-loaded autologous monocytes (Figure 1a). 

**Figure 2:**
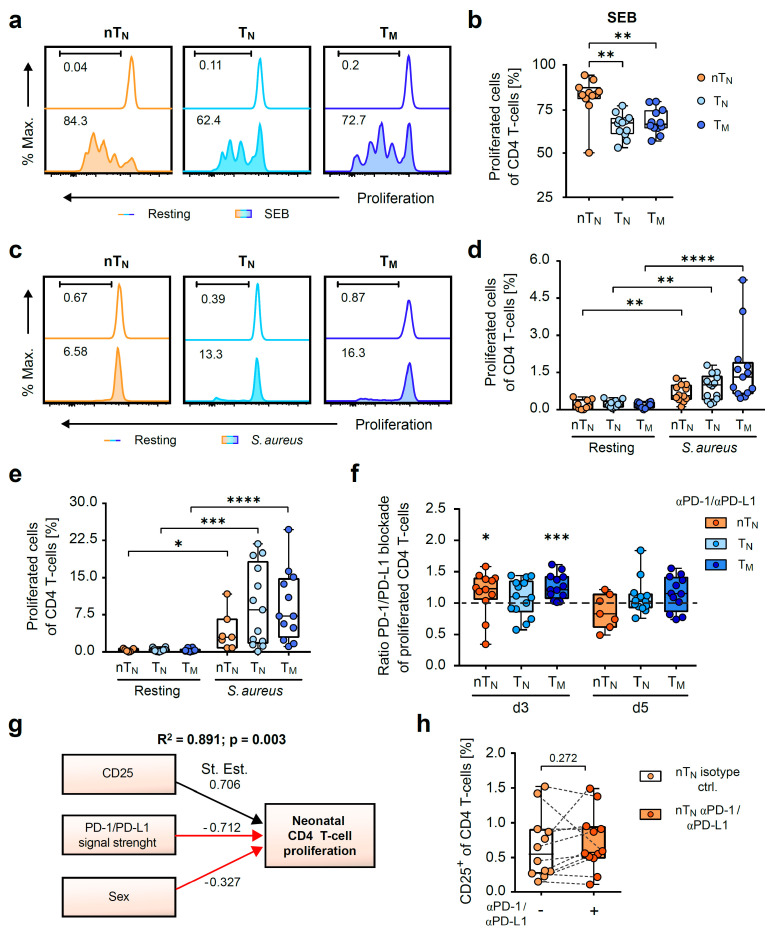
PD-1/PD-L1 blockade enhances early anti-*S. aureus*-initiated proliferation in neonatal naïve and adult memory CD4 T-cells. (**a**) Representative histograms of the proliferation of neonatal naïve (nT_N_) or adult naïve (T_N_) and memory (T_M_) CD4 T-cells in resting conditions or in response to SEB on day 3 (**b**) Frequencies of proliferated nT_N_, T_N_ or T_M_ on day 3 after SEB activation. (**c**) Representative proliferation histograms of nT_N_, T_N_ or T_M_ in resting conditions and in response to *S. aureus* on day 5 (**d**,**e**) Frequencies of proliferated nT_N_, T_N_ or T_M_ in resting or *S. aureus*-mediated activating conditions on day 3 (**d**) or day 5 (**e**). (**f**) Ratios of the proliferation of *S. aureus*-stimulated PD-1/PD-L1-blocked CD4 T-cells to their isotype controls. The significance between the blockade and the isotype control is depicted above the respective box plot. (**g**) Path diagram of a linear regression analysis of factors (coefficients) regulating anti-*S. aureus*-specific proliferation in nT_N_ (sex: female was coded 1, male 2). (**h**) Frequencies of CD25^+^ nT_N_ on day 3 after activation with *S. aureus* under PD-1/PD-L1 blockade or isotype control conditions. Data points represent donors in box plots with median, interquartile, and range. Numbers indicate the percentage of proliferated T-cells, *p*-values, adjusted R^2^, or standardized estimates; * *p* < 0.05, ** *p* < 0.01, *** *p* < 0.001, **** *p* < 0.0001; *p*-values were calculated using the Kruskal–Wallis test (**b**,**d**,**e**), paired *t*-test, Wilcoxon test (**f**,**h**), or linear regression analysis (**g**).

First, the polyclonal T-cell proliferation induced by the mitogen SEB was analyzed by the division-induced dilution of CFSE (Figure 2a). In this regard, nT_N_ showed cell divisions (CFSE^low^, median 83.3), that were stronger than those of T_N_ (median 67.1) and T_M_ (median 66.4) (Figure 2b). 

By analyzing the antigen-specific proliferation against *S. aureus* we clearly observed an expansion in all the CD4 T-cell subpopulations analyzed as early as day 3, which intensified on day 5 after the onset of stimulation (Figure 2c–e). Next, the sensitivity of the proliferative response to the PD-1/PD-L1 blockade was assessed during the stimulation by *S. aureus*. As a result, the early susceptibility of neonatal naïve and adult memory CD4 T-cells was revealed by the ratio of PD-1/PD-L1-blocked *S. aureus*-stimulated CD4 T-cells to *S. aureus*-stimulated control T-cells (Figure 2f).

These data imply that proliferation is controlled by PD-1 in neonates and adults during less stringent, antigen-specific conditions. Our data show that there are different proliferation rates in terms of the speed and number of proliferating T-cells in the neonatal and adult subpopulations (Figure 2a–e). To analyze the role of antibacterial proliferation of the neonatal T-cell responses, we performed a regression analysis on all neonatal responses (Figure 2g). These results depicted in a model (adjusted R^2^ = 0.891, *p* = 0.003) show that in addition to sex, the extent of the PD-1/PD-L1 blockade and the frequency of CD25 expression in response to *S. aureus* have a highly significant impact on the actual proliferation rate of anti-*S. aureus*-specific neonatal T-cells. The blockade of PD-1 and PD-L1 did not significantly increase the frequencies of CD25-expressing neonatal CD4 T-cells. However, the linear regression analysis revealed that this relationship significantly determines the magnitude of the proliferative response to *S. aureus* of the respective donor. Of note, CD69 expression of nTN poorly correlates (R^2^ = 0.387, *p* = 0.041) with proliferation when applying Pearson’s correlation and addition to the linear regression analysis did not improve the model. Further, the gestational age of the individuals did not play a significant role in our model. Thus, sex (female) and the ability to block PD-1 appear to be key players in anti-*S. aureus*-directed CD4 T-cell responses.

### 2.3. PD-1/PD-L1 Blockade Increases the Magnitude of S. aureus-Antigen-Specific T-Helper Cells in Neonates

**Figure 3:**
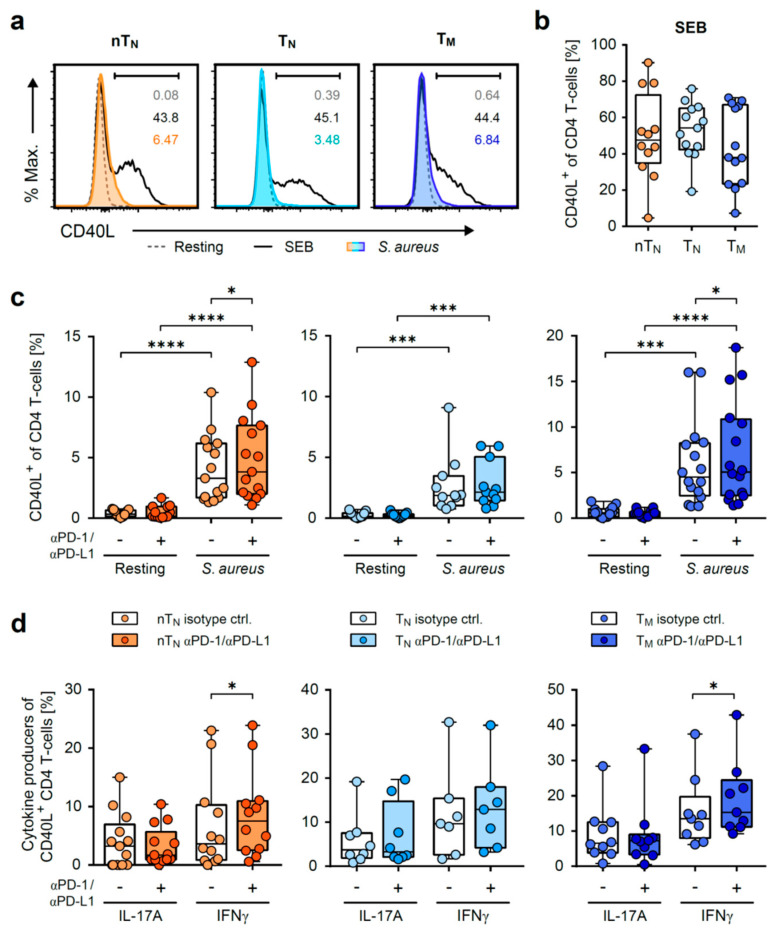
PD-1/PD-L1 signals regulate antigen-specific CD4 T-cell responses of neonatal naïve and adult memory T-helper cells. (**a**) Representative histograms of the CD40L expression of *S. aureus* and SEB-activated, or resting nT_N_, T_N_ or T_M_ on d3. (**b**) Frequencies of CD40L^+^ nT_N_, T_N_ or T_M_ on day 3 after SEB-activation. (**c**) CD40L expression of resting or *S. aureus*-activated nT_N_ (left), T_N_ (middle) or T_M_ (right) on day 3, treated with PD-1/PD-L1-blocking or isotype control antibodies. (**d**) Frequencies of IL-17A or IFNγ producers of *S. aureus*-activated CD40L^+^ nT_N_, T_N_ or T_M_ on day 3, treated with PD-1/PD-L1-blocking or isotype control antibodies. Data points represent donors in box plots with median, interquartile, and range. Numbers indicate the percentage of CD40L^+^ cells; * *p* < 0.05, *** *p* < 0.001, **** *p* < 0.0001; *p*-values were calculated using two-way ANOVA (Fisher’s LSD) (**c**) or the Wilcoxon test (**d**).

To discriminate between the effects of the PD-1/PD-L1 blockade on antigen-specific responding T-helper cells and the possible effects on the indirect activation of the CD4 T-cell pool by unspecific means, such as bystander activation, we monitored CD40L expression that was exclusively up-regulated by TCR triggering [28]. Neonatal and adult T-helper cells consequently showed a strong up-regulation of CD40L expression upon polyclonal activation with a SEB superantigen, whilst *S. aureus*-activated T-cells also robustly expressed CD40L and resting T-cells remained CD40L^low^ (Figure 3a–c). Of note, T-cell subsets stimulated with SEB showed no differences in CD40L expression (Figure 3b). Intriguingly, the PD-1/PD-L1 blockade significantly increased CD40L expression in *S. aureus*-activated nT_N_, similarly to T_M_, whereas no effect of the blocking antibodies could be detected in T_N_ (Figure 3c).

Next, the IFNγ and IL-17A production of CD40L^+^ T cells was assessed for functional characterization of the antigen-specific activated T cells. By using the activation system of *S. aureus*-loaded monocytes, we could predominantly provoke Th1-like responses as the percentages of IL-17A-positive T-cells remained lower than those of IFNγ-positive T-cells in all groups (Figure 3d). As a result of the antibody-mediated blockade of PD-1 and PD-L1, IFNγ production strongly increased in CD40L^+^ neonatal T-helper cells (median from 3.6 to 7.5) and a similar effect was observed in adult memory, but not naïve, T-helper cells (Figure 3d).

Hence, the analysis of CD40L-expressing CD4 T-cells revealed that the PD-1/PD-L1 axis controls both the magnitude and functionality of *S. aureus*-specifically activated naïve neonatal and adult memory T-helper cells.

### 2.4. PD-1/PD-L1 Blockade Increases the Frequency of S. aureus-Induced Inflammatory Cytokine Producers in Neonatal T-Helper Cells

**Figure 4:**
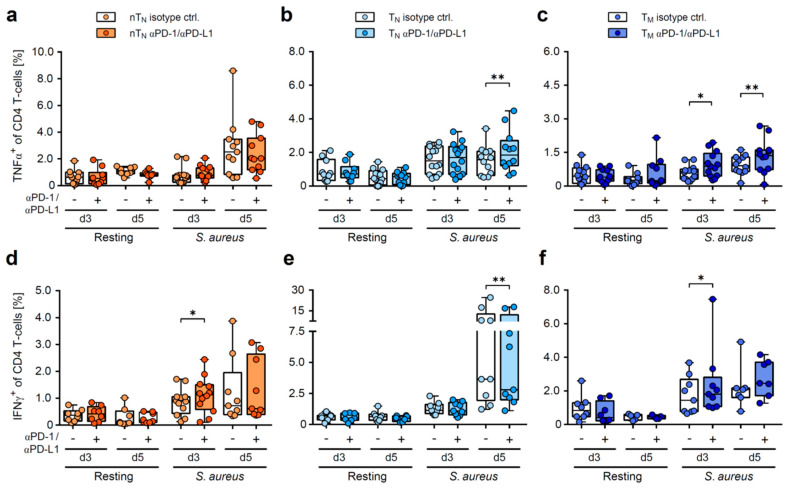
PD-1/PD-L1 axis regulates the early IFNγ response of neonatal CD4 T-cells. Frequencies of TNFα (**a**–**c**) or IFNγ (**d**–**f**) producers of resting or *S. aureus*-activated nT_N_ (**a**,**d**), T_N_ (**b**,**e**) or T_M_ (**c**,**f**) on day 3 and day 5, treated with PD-1/PD-L1-blocking or isotype control antibodies as indicated. Data points represent donors in box plots with median, interquartile, and range. * *p* < 0.05, ** *p* < 0.01; *p*-values were calculated using two-way ANOVA (Fisher’s LSD).

To extend the investigation of the age-related effects of the PD-1 blockade on the functionality of T-helper cells from neonates and adults, the early and late expression of the Th1-like cytokines (TNFα, IFNγ) of total CD4 T-cells was analyzed by flow cytometry (Figure 4a–f). Therefore, CD4 T-cell compartments from neonates and adults were again incubated with anti-PD-1 and anti-PD-L1 antibodies or an isotype control, respectively, activated with autologous monocytes matured overnight with h.i. *S. aureus*, and analyzed on day 3 and day 5. 

Of note, no effect of the PD-1/PD-L1 blockade on the frequencies of either TNFα, or IFNγ-producing T-cells could be detected under resting conditions (Figure 4a–f). However, upon *S. aureus*-specific stimulation, increased frequencies of TNFα-producing adult CD4 T-cells were observed, whereas TNFα production in the neonatal T-helper cells remained unaffected (Figure 4a–c). Instead, IFNγ expression was significantly increased in the neonatal T-cells as early as day 3 when PD-1/PD-L1 was blocked during stimulation, but not in their adult naïve counterparts (Figure 4d,e). Again, similar to neonatal T-helper cells, the adult memory CD4 T-cell compartment exclusively showed an early impact of the PD-1/PD-L1 axis in this regard (Figure 4f). Of note, adult naïve CD4 T-cells were also sensitive to the PD-1/PD-L1 blockade upon stimulation, but this only appeared to be the case for later cytokine expression (Figure 4b,e). Together, these results show a specific regulation of the *S. aureus*-induced pro-inflammatory cytokine response by the PD-1/PD-L1 axis in neonates that is restricted on the immediate production of IFNγ. Our data also show that neonatal T-cells have properties that are similar to those of memory T-cells from adults.

**Figure 5:**
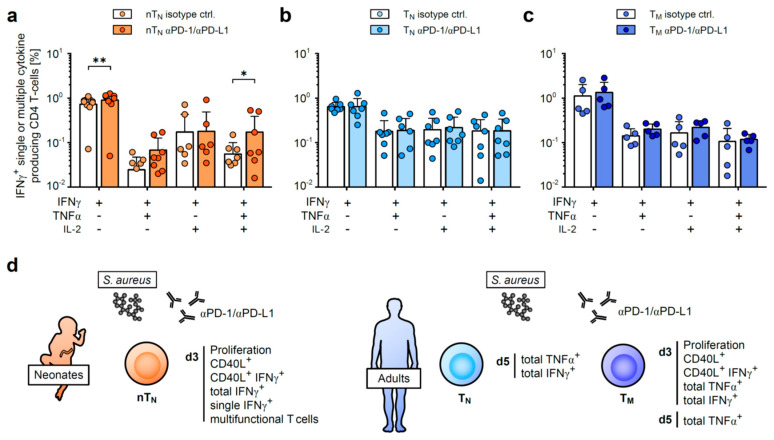
Multi-functionality of neonatal CD4 T-cells is controlled by PD-1/PD-L1. (**a**–**c**) IFNγ single or IFNγ co-expression along TNFα and/or IL-2 in nT_N_ (**a**), T_N_ (**b**) or T_M_ (**c**) on day 3 in response to *S. aureus*. (**d**) Summary of PD-1/PD-L1-regulated functions in *S. aureus*-activated neonatal naïve (nT_N_, left) or naïve (T_N_) and memory (T_M_) CD4 T-cells from adults (right). Data points represent donors in bar graphs with mean and SD. * *p* < 0.05, ** *p* < 0.01; *p*-values were calculated using two-way ANOVA (Fisher’s LSD).

Th1 cells, which are capable of producing multiple cytokines simultaneously, are considered as high-quality effector cells that provide a potent defense against bacterial and viral infections [29]. Therefore, we next analyzed the age-dependent effect of PD-1/PD-L1 signals on the variability of antigen-specific T-cell multi-functionality. The majority of neonatal anti-*S. aureus*-activated Th1 cells were demonstrated to be IFNγ single producers (Figure 5a). The blockade of PD-1/PD-L1 increased the proportion of these cells, whereas no effect on neither IFNγ single, nor co-producers, could be observed in the adult T-cell compartments (Figure 5a–c). 

Intriguingly, triple-cytokine producers were detectable in all the *S. aureus*-stimulated T-cell subpopulations; however, only neonatal CD4 T-cells were susceptible to PD-1/PD-L1 inhibition (Figure 5a–c). These data further underline that neonatal T-cell responses are more stringently controlled by the PD-1/PD-L1 axis than those of adults.

In summary, this study underlines the distinct need for the PD-1/PD-L1-mediated regulation of anti-microbial T-helper cell responses in neonates vs. adults. Naïve neonatal CD4 T-cell responses are immediately regulated by the PD-1/PD-L1 axis in terms of proliferation, antigen-specificity and functionality, rather resembling the regulation of adult memory CD4 T-cells in this regard, compared to their naïve adult counterparts (Figure 5d). 

## 3. Discussion

In this study, we show for the first time that the functionality of the neonatal antibacterial T-helper cell response is controlled by the immune-checkpoint molecules PD-1 and its ligand PD-L1. The comparison of the sensitivities of neonatal and adult CD4 T-cells to the PD-1/PD-L1 blockade supports the growing notion that neonatal T-cells are not inherently defective, but rather susceptible to inhibitory signals [11]. As CD25^+^ T-cells were eliminated prior to stimulation, it is unlikely that pre-existing nTreg cells (FoxP3, CD25^high^) induced an *S. aureus*-mediated conversion of the conventional T-helper cells that could be controlled by the PD-1/PD-L1 axis [30]. 

Several functions of neonatal CD4 T-cells analyzed in this study are in accordance with the needs of neonatal adaptive immune responses, that are a necessity of immediate functional defense with no precipitous memory formation [11]. In line with this, our results on SEB-activated neonatal T cells demonstrate their ability to mount strong responses for immediate pathogen control early in life. In contrast to other studies that have suggested the innate-like properties of neonatal T-cells [31], our results point towards properties of neonatal T-cells resembling the regulation of memory T-cells. Consequently, the neonatal T-helper-cell response is immediately controlled by the PD-1/PD-L1 axis to prevent pathologies caused by CD4 T-cell hyperreactivity during the development of newborns. We therefore postulate that in a suddenly changing environment, due to birth, where immunologic memory is scarce, these neonatal cells acquire tightly regulated memory-like functions in order to provide a proper anti-bacterial defense while ensuring tolerance to harmless antigens. Since neonatal naïve T-cells were stimulated in the absence of memory and regulatory T-cells, it is likely that the neonatal conventional CD4 T-cells exert this function intrinsically. Hence, our data indicate that these T-cells could act as a distinct population of lymphocytes well suited to the demands of early life.

Our regression model (Figure 2g) reveals that sex has a significant influence on the antigen-specific cellular response of neonates to the model pathogen *S. aureus*, with male neonates showing reduced T-cell proliferation. The different levels of sex hormones in male and female individuals may explain sex-specific adaptive immune responses in neonates, as is known to be the case for adults [32]. Sex differences in the adult infection defense are well established, e.g., in SARS-CoV-2 [33] or bacterial infections [34]. Our data may therefore help to explain why female neonates show decreased mortality compared to males, due to a more rigorous immune response [35,36]. Sex hormones, such as testosterone or estrogen, are already produced during embryonic development and could increase the expression and thus the inhibitory potential of immune checkpoint inhibitors [37].

So far, the regulation of the antigen-specific responses of neonatal conventional CD4 T-cells by immune checkpoint molecules has hardly been studied but is known to be central to adult responses [38]. In this context, TLR signaling as a co-activator of neonatal T-cells [39,40] could be potentially involved in an indirect manner despite removing residual *S. aureus* TLR-ligands from APCs prior to the activation of T-cells. However, inhibiting the major TLR-signaling pathway molecule MyD88 using a chemical inhibitor did not affect T-cell activation in similar setting of antigen-specific stimulation [21]. In conclusion, our findings on the PD-1/PD-L1-regulated functionality of neonatal T-cell responses reveal novel insights into the distinct impact of immune-checkpoint molecules on these T-cells and underline that neonatal naïve T-cells act as an independent, well-adapted lineage apart from their adult counterparts.

## 4. Materials and Methods

### 4.1. Samples

The umbilical cord blood samples were obtained from the umbilical cords of 15 term-born newborns (6 females, 8 males, 1 N/A) immediately after birth by vaginal delivery supplied by the hospital St. Marienstift in Magdeburg, Germany. The gestational age ranged between 38/3 and 41/3 (median gestational age: 39/6). Blood samples from 17 healthy adult donors (2 females, 15 males, age distribution between 20 and 66 years, median age: 47 years) were provided by the Institute of Transfusion Medicine and Immunohematology at the University Hospital of Magdeburg from leukocyte reduction filters (Asahi Kasei Medical Co., Tokyo, Japan). 

### 4.2. Cell Isolation

The umbilical cord blood mononuclear cells (CBMCs) and adult peripheral blood mononuclear cells (PBMCs) were prepared by density gradient centrifugation using Pancoll (PAN BioTech, Aidenach, Germany). The CBMCs were centrifuged at 400× *g* and the PBMCs at 1000× *g*. Subsequently, the erythroblasts of the CBMCs were lysed with BD Pharm Lyse buffer (BD Biosciences, Franklin Lakes, NJ, USA). The adult and neonatal CD14^+^ monocytes were isolated from the purified PBMCs and CBMCs, respectively, by magnetic bead separation using CD14 MicroBeads and the autoMACS Pro Separator (Miltenyi Biotec, Bergisch Gladbach, Germany) according to the manufacturer’s instructions. The naïve neonatal CD4 T-cells (nT_N_) were isolated by depletion (Naïve CD4 T-cell Isolation Kit 2; Miltenyi Biotec) according to the manufacturer’s protocol. The adult naïve CD45RA^+^ CD4 T-cells (T_N_) and effector/memory CD45RO^+^ CD4 T-cells (T_M_) were separated in two consecutive steps. First, the Treg-depleted CD4 T-cells were purified using the CD4 T-cell Isolation Kit (Miltenyi Biotec) with the additional use of a biotinylated anti-CD25 antibody (BC96; Biolegend, San Diego, CA, USA). Second, the T_N_ were separated by the depletion of the T_M_ using CD45RO MicroBeads, and vice versa, the T_M_ were isolated by the depletion of the T_N_ using CD45RA MicroBeads (both from Miltenyi Biotec). The purities of the isolated cell fractions were routinely assessed by flow cytometry. In the case of insufficient amounts of nT_N_, not all conditions were tested simultaneously, resulting in different sample sizes.

### 4.3. Cell Culture and Cell Activation

The monocytes were matured with h.i. *S. aureus* with no expression of the Staphylococcal enterotoxin B (SEB^neg^) [25]. In brief, the monocytes were pulsed with 10 µg/mL *S. aureus* in a complete RPMI1640 medium (PAN BioTech) for 24 h at 37 °C/5% CO_2_. An LPS analysis revealed a concentration lower than 0.04 EU/mL (Pierce LAL Chromogenic Endotoxin Quantitation Kit, Thermo Fisher Scientific, Waltham, MA, USA). Prior to co-cultivation with autologous CD4 T-cells, the monocytes were thoroughly washed. For the antigen-specific T-cell activation, the CD4 T-cells were co-cultured with *S. aureus*-pulsed monocytes at a ratio of 2.4:1 (neonatal cells) or 2:1 (adult cells) for 3 or 5 days. Additionally, the CD4 T-cells were polyclonal activated by adding 1 µg/mL of SEB (Sigma-Aldrich/Merck, Darmstadt, Germany) or cultivated unstimulated with non-pulsed monocytes, respectively. The PD-1/PD-L1 on CD4 T-cells was blocked by adding 10 µg/mL of an anti-PD-1 antibody (EH12.2; BD Biosciences) and 10 µg/mL of an anti-PD-L1 antibody (MIH1; BD Biosciences) or IgG1, κ isotype control antibody (107.3; BD Biosciences).

### 4.4. Flow Cytometric Analysis

Cell proliferation, viability, the expression of activation markers and the cytokine production of neonatal and adult CD4 T-cells were analyzed by flow cytometry on day 3 and 5 after the start of stimulation. To determine cell proliferation, CD4 T-cells were labeled with the vital dye carboxyfluorescein succinimidyl ester (CFSE; Thermo Fisher Scientific) and the CFSE dilution was measured. Prior to staining, the CD4 T-cells were treated with 5 µg/mL of Brefeldin A (Cell Signaling Technology Inc., Danvers, MA, USA) for 4h and with 10 ng/mL of PMA (Sigma-Aldrich/Merck) and 1 µg/mL of ionomycin (Cell Signaling Technology) in the last hour. The cell surface receptors of nT_N_, T_N_ and T_M_ were stained with the following fluorescent labeled antibodies: anti-CD4 (REA623; Miltenyi Biotec), anti-CD3 (SK7; Biolegend), anti-CD25 (BC96; Biolegend), anti-CD69 (FN50; Biolegend) and anti-PD-1 (EH12.2H7; Biolegend). For intracellular staining, the cells were fixed and permeabilized with 0.5% saponine (Sigma-Aldrich/Merck). The following antibodies were used: anti-TNFα (Mab11; Biolegend), anti-IFNγ (4S.B3; BD Biosciences), anti-IL-2 (MQ1-17H12; Biolegend), anti-IL17A (BL168; Biolegend) and anti-CD40L (CD154) (REA238; Miltenyi Biotec). Viable cells were determined according to Zombie dye (Biolegend) negative staining. All flow cytometric measurements were performed using the FACS-Canto II (BD Biosciences) and BD FACSDiva software v9.0.1 (BD Biosciences). The data was then further analyzed with FlowJo software v10.7.1 (BD Biosciences).

### 4.5. Statistical Analysis

The statistical analysis was performed using the GraphPad Prism 8 software (GraphPad Software, Boston, MA, USA). Outliers determined by the Grubbs test were excluded. The data are presented as boxplots showing the 25% quartile or mean ± SD. The normal distribution of data sets was tested with the Shapiro–Wilk test. The significance analysis was performed using the paired *t*-test or the Wilcoxon matched-pairs signed-rank test. The Kruskal–Wallis test with Dunn’s multiple comparison test, one-way ANOVA with Sidak’s multiple comparison test, or two-way ANOVA with Fisher’s LSD test were used for multiple comparisons. Statistical significance was reached when *p* < 0.05 (*), *p* < 0.01 (**), *p* < 0.001 (***) and *p* < 0.0001 (****). The interrelationship between variables was analyzed by multiple linear regression with Jamovi software v2.3.21 (The jamovi project 2022, Sydney, Australia).

## Data Availability

The data presented in this study are available on request from the corresponding author. The data are not publicly available due to ethical restrictions.

## References

[1] World Health Organization Newborn Infections. https://www.who.int/teams/maternal-newborn-child-adolescent-health-and-ageing/newborn-health/newborn-infections.

[2] Filleron A., Beauregard-Birba S., Mura T., Aujoulat F., Michon A.L., Rodière M., Tran T.A., Jeziorski E., Marchandin H. (2018). Survey of Staphylococcus aureus in a general pediatric population and focus on isolates with three clinically relevant toxin-encoding genes. World J. Pediatr..

[3] Ondusko D.S., Nolt D. (2018). *Staphylococcus* *aureus*. Pediatr. Rev..

[4] Kaplan S.L. (2016). Staphylococcus aureus Infections in Children: The Implications of Changing Trends. Pediatrics.

[5] Antimicrobial Resistance Collaborators (2022). Global burden of bacterial antimicrobial resistance in 2019: A systematic analysis. Lancet.

[6] Pierau M., Arra A., Brunner-Weinzierl M.C. (2021). Preventing Atopic Diseases During Childhood—Early Exposure Matters. Front. Immunol..

[7] Fujimura K.E., Sitarik A.R., Havstad S., Lin D.L., LeVan S., Fadrosh D., Panzer A.R., LaMere B., Rackaityte E., Lukacs N.W. (2016). Neonatal gut microbiota associates with childhood multisensitized atopy and T cell differentiation. Nat. Med..

[8] Risnes K.R., Belanger K., Murk W., Bracken M.B. (2010). Antibiotic Exposure by 6 Months and Asthma and Allergy at 6 Years: Findings in a Cohort of 1,401 US Children. Am. J. Epidemiol..

[9] Shaw S.Y., Blanchard J.F., Bernstein C.N. (2010). Association Between the Use of Antibiotics in the First Year of Life and Pediatric Inflammatory Bowel Disease. Am. J. Gastroenterol..

[10] Kronman M.P., Zaoutis T.E., Haynes K., Feng R., Coffin S.E. (2012). Antibiotic Exposure and IBD Development Among Children: A Population-Based Cohort Study. Pediatrics.

[11] Rudd B.D. (2020). Neonatal T Cells: A Reinterpretation. Annu. Rev. Immunol..

[12] Mold J.E., Venkatasubrahmanyam S., Burt T.D., Michaëlsson J., Rivera J.M., Galkina S.A., Weinberg K., Stoddart C.A., McCune J.M. (2010). Fetal and Adult Hematopoietic Stem Cells Give Rise to Distinct T Cell Lineages in Humans. Science.

[13] Schaub B., Liu J., Höppler S., Schleich I., Huehn J., Olek S., Wieczorek G., Illi S., von Mutius E. (2009). Maternal farm exposure modulates neonatal immune mechanisms through regulatory T cells. J. Allergy Clin. Immunol..

[14] Schmiedeberg K., Krause H., Röhl F.-W., Hartig R., Jorch G., Brunner-Weinzierl M.C. (2016). T Cells of Infants Are Mature, but Hyporeactive Due to Limited Ca2+ Influx. PLoS ONE.

[15] Li G., Yu M., Lee W.-W., Tsang M., Krishnan E., Weyand C.M., Goronzy J.J. (2012). Decline in miR-181a expression with age impairs T cell receptor sensitivity by increasing DUSP6 activity. Nat. Med..

[16] Hebel K., Weinert S., Kuropka B., Knolle J., Kosak B., Jorch G., Arens C., Krause E., Braun-Dullaeus R.C., Brunner-Weinzierl M.C. (2014). CD4+ T Cells from Human Neonates and Infants Are Poised Spontaneously to Run a Nonclassical IL-4 Program. J. Immunol..

[17] Halonen M., Lohman I.C., Stern D.A., Spangenberg A., Anderson D., Mobley S., Ciano K., Peck M., Wright A.L. (2009). Th1/Th2 Patterns and Balance in Cytokine Production in the Parents and Infants of a Large Birth Cohort. J. Immunol..

[18] Gibbons D., Fleming P., Virasami A., Michel M.-L., Sebire N., Costeloe K., Carr R., Klein N., Hayday A. (2014). Interleukin-8 (CXCL8) production is a signatory T cell effector function of human newborn infants. Nat. Med..

[19] Hebel K., Rudolph M., Kosak B., Chang H.-D., Butzmann J., Brunner-Weinzierl M.C. (2011). IL-1β and TGF-β Act Antagonistically in Induction and Differentially in Propagation of Human Proinflammatory Precursor CD4+ T Cells. J. Immunol..

[20] Razzaghian H.R., Sharafian Z., Sharma A.A., Boyce G.K., Lee K., Da Silva R., Orban P.C., Sekaly R.-P., Ross C.J., Lavoie P.M. (2021). Neonatal T Helper 17 Responses Are Skewed Towards an Immunoregulatory Interleukin-22 Phenotype. Front. Immunol..

[21] Vogel K., Pierau M., Arra A., Lampe K., Schlueter D., Arens C., Brunner-Weinzierl M.C. (2018). Developmental induction of human T-cell responses against Candida albicans and Aspergillus fumigatus. Sci. Rep..

[22] Jubel J.M., Barbati Z.R., Burger C., Wirtz D.C., Schildberg F.A. (2020). The Role of PD-1 in Acute and Chronic Infection. Front. Immunol..

[23] Dietz S., Molnar K., Riedel H., Haag L., Spring B., Orlikowsky T.W., Poets C.F., Gille C., Köstlin-Gille N. (2022). Expression of immune checkpoint molecules on adult and neonatal T-cells. Immunol. Res..

[24] Titov A., Kaminskiy Y., Ganeeva I., Zmievskaya E., Valiullina A., Rakhmatullina A., Petukhov A., Miftakhova R., Rizvanov A., Bulatov E. (2022). Knowns and Unknowns about CAR-T Cell Dysfunction. Cancers.

[25] Arra A., Pech M., Fu H., Lingel H., Braun F., Beyer C., Spiliopoulou M., Bröker B.M., Lampe K., Arens C. (2021). Immune-checkpoint blockade of CTLA-4 (CD152) in antigen-specific human T-cell responses differs profoundly between neonates, children, and adults. Oncoimmunology.

[26] Sharma A.A., Jen R., Butler A., Lavoie P.M. (2012). The developing human preterm neonatal immune system: A case for more research in this area. Clin. Immunol..

[27] Bae J.S., Da F., Liu R., He L., Lv H., Fisher E.L., Rajagopalan G., Li M., Cheung G.Y.C., Otto M. (2021). Contribution of Staphylococcal Enterotoxin B to *Staphylococcus aureus* Systemic Infection. J. Infect. Dis..

[28] Splawski J.B., Nishioka J., E Lipsky P. (1996). CD40 ligand is expressed and functional on activated neonatal T cells. J. Immunol..

[29] Darrah P.A., Patel D.T., De Luca P.M., Lindsay R.W.B., Davey D.F., Flynn B.J., Hoff S.T., Andersen P., Reed S.G., Morris S.L. (2007). Multifunctional TH1 cells define a correlate of vaccine-mediated protection against Leishmania major. Nat. Med..

[30] Rabe H., Nordström I., Andersson K., Lundell A., Rudin A. (2014). *Staphylococcus aureus* convert neonatal conventional CD4(+) T cells into FOXP3(+) CD25(+) CD127(low) T cells via the PD-1/PD-L1 axis. Immunology.

[31] Siefker D.T., Adkins B. (2016). Rapid CD8+ Function Is Critical for Protection of Neonatal Mice from an Extracellular Bacterial Enteropathogen. Front. Pediatr..

[32] Klein S.L., Flanagan K.L. (2016). Sex differences in immune responses. Nat. Rev. Immunol..

[33] Takahashi T., Ellingson M.K., Wong P., Israelow B., Lucas C., Klein J., Silva J., Mao T., Oh J.E., Tokuyama M. (2020). Sex differences in immune responses that underlie COVID-19 disease outcomes. Nature.

[34] Vázquez-Martínez E.R., García-Gómez E., Camacho-Arroyo I., González-Pedrajo B. (2018). Sexual dimorphism in bacterial infections. Biol. Sex Differ..

[35] Drevenstedt G.L., Crimmins E.M., Vasunilashorn S., Finch C.E. (2008). The rise and fall of excess male infant mortality. Proc. Natl. Acad. Sci. USA.

[36] Federal Institute for Population Research Sex Dependent Mortality of Infants in Germany (1990–2020). https://www.bib.bund.de/Permalink.html?cms_permaid=1217856.

[37] Luna R.M.L., Körmendy D., Brunner-Weinzierl M.C. (2010). Female-biased incidence of experimental autoimmune encephalomyelitis reflects sexually dimorphic expression of surface CTLA-4 (CD152) on T lymphocytes. Gend. Med..

[38] Körmendy D., Hoff H., Hoff P., Bröker B.M., Burmester G.-R., Brunner-Weinzierl M.C. (2013). Impact of the CTLA-4/CD28 axis on the processes of joint inflammation in rheumatoid arthritis. Arthritis Rheum..

[39] Komai-Koma M., Jones L., Ogg G.S., Xu D., Liew F.Y. (2004). TLR2 is expressed on activated T cells as a costimulatory receptor. Proc. Natl. Acad. Sci. USA.

[40] Sinnott B.D., Park B., Boer M.C., Lewinsohn D.A., Lancioni C.L. (2016). Direct TLR-2 Costimulation Unmasks the Proinflammatory Potential of Neonatal CD4+ T Cells. J. Immunol..

